# Understanding and rationalizing the structure of ionic solids by computational application of Beck’s extended coordination number rule

**DOI:** 10.1107/S2052520626001794

**Published:** 2026-04-01

**Authors:** Niklas Langer, Holger Kohlmann

**Affiliations:** a Institute of Inorganic Chemistry and Crystallography, Faculty of Chemistry Leipzig University Johannisallee 29 Leipzig 04103 Germany; University of Geneva, Switzerland

**Keywords:** coordination number rule, crystal chemistry, ionic solids

## Abstract

Crystal chemical rules are widely applied to understand and rationalize inorganic crystal structures. Examples of such include Pauling rules and Beck’s extended coordination number rule. For the latter no large-scale benchmark has been performed yet. A rigorous analysis of the validity of the extended coordination number rule was performed on a dataset consisting of 1038 ternary fluorides. 59.9% of the examined crystal structures agree with Beck’s rule. An additional 22.3% of structures the deviations can be explained by a more or equally homogenous distribution of charges, thus yielding a total rationalization power of 82.2%. The structural complexity of the crystal structures was identified as a main reason for disagreement with Beck’s rule.

## Abstract 2

Crystal chemical rules are powerful tools to understand and rationalize inorganic crystal chemical structures. Using an automated approach, Beck’s extended coordination number rule is proven to correctly rationalize the coordination within 82.2% of all ternary fluorides.

## 1. Introduction

The design of new materials is crucial in tackling the economic and environmental challenges of this century. To explore and design novel chemical compounds it is often helpful to first understand the structures of the ones that are already known. For this purpose of explaining and rationalizing the structures of inorganic solids, crystal chemical rules are widely used. This work is going to focus on understanding the coordination in ionic solids. The structures of such compounds are dominated by electrostatic interactions instead of covalent bonds. The interatomic connectivity in this type of structures can be described by coordination numbers (CNs) and coordination polyhedra. These concepts aim to describe the immediate surrounding of an atom by specifying the number of the closest neighboring atoms and their geometric arrangement (Müller, 2006 ▸; Müller, 2008 ▸). Despite the simplicity of this concept, the determination of CNs can be challenging when the distances to the neighboring atoms are very similar. This gave rise to a multitude of different methods for CN determination, which are generally based either on interatomic distances or on a Voronoï tesselation. The former includes the procedure based on the largest gap in the histogram of interatomic distances developed by Brunner (1977 ▸) and the ECoN algorithm proposed by Hoppe (1979 ▸), whereas the latter encompasses the methods proposed by O’Keeffe (1979 ▸), the algorithm by Waroquiers *et al.* (2020 ▸) included in the ChemEnv library and the CrystalNN method by Pan *et al.* (2021 ▸). A benchmark of the most important methods is readily available in the literature (Pan *et al.*, 2021 ▸). The procedure that was ultimately chosen for this investigation was the reciprocal largest gap method by Brunner. All methods that were tested in the scope of this publication and the reasons for choosing this specific procedure for CN determination are presented in Section 3.2.

Since the structures of ionic solids consist of interconnected coordination polyhedra, understanding of the coordination within a structure also fosters understanding of the structure itself and its structure–property relationships. The most well known crystal chemical rules for ionic compounds are Pauling’s rules (Pauling, 1929 ▸):

(i) *Radius-ratio rule:* ‘A coordination polyhedron of anions is formed around every cation. The cation–anion distances are determined by the sum of the ionic radii, and the coordination number of the cation by the radius ratio’ (Müller, 2006 ▸; Müller, 2008 ▸).

(ii) *Electrostatic-valence rule:* ‘In a stable ionic structure the valence (ionic charge) of each anion with changed sign is exactly or nearly equal to the sum of the electrostatic bond strengths to it from adjacent cations. The electrostatic bond strength is defined as the ratio of the charge on a cation to its coordination number’ (Müller, 2006 ▸; Müller, 2008 ▸).

(iii) *The sharing of edges and faces:* ‘The presence of shared edges and especially of shared faces in a structure decreases its stability; this effect is large for cations with high charge and low coordination number’ (Müller, 2006 ▸; Müller, 2008 ▸).

(iv) *The nature of contiguous polyhedra:* ‘In a crystal containing different cations, those with large charge and small coordination number tend not to share polyhedron elements with each other, *i.e.* they tend to keep as far apart as possible’ (Müller, 2006 ▸; Müller, 2008 ▸).

(v) *The rule of parsimony:* ‘The number of essentially different kinds of constituents in a crystal tends to be small’ (Pauling, 1929 ▸).

Recent publications have assessed the predictive power of Pauling’s rules on a dataset consisting of 5000 oxides. It has been shown that each of the rules by itself shows an agreement with the experimentally determined structures of about 60–70% (with the exception of the electrostatic-valence rule that only appears to be valid for highly symmetrical, undistorted coordination environments). However, only around 13% of the investigated compounds fulfill at least four of Pauling’s rules combined (George *et al.*, 2020 ▸). This means that each of Pauling’s rules is able to rationalize one aspect of the coordination in an ionic structure but only for very few structures do all these aspects hold true simultaneously. A different approach to the rationalization of the coordination in ionic solids is given by the *extended coordination number rule* proposed by Beck (2014 ▸). This crystal chemical rule does not focus on describing a part of the coordination within a structure but aims on rationalizing the coordination of all atoms at once. The basis of the CN rule is the trivial truth that in ionic compounds the sum of the CNs of the cations must be equal to the sum of the CNs of the anions. For a ternary compound *A*_*a*_^[*p*]^*B*_*b*_^[*q*]^*X*_*x*_^[*r*]^with integer CNs this is expressed by
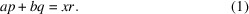


If this equation holds true, one should be able to calculate the coordination of the anions just using the coordination of the cations and vice versa. For this to work a clear classification into anions and cations is necessary, which makes an application of Beck’s rule for metals or covalent structures not straightforward. This has been tested positively, but only on very few examples (Voss & Kohlmann, 2017 ▸), which is why this publication will focus on salt-like compounds. In the way Beck proposed this theory, the CNs of the cations are used as input for the calculations which then yield the coordination environments of the anions and therefore the coordination within the whole structure. The calculations follow the *principle of simplicity* with the simplest coordination being given by the *simplest numerical solution* (SNS). For a compound as simple as described in equation (1) this solution is mostly trivial, but in most cases a ternary compound will show multiple anionic sites with different coordination environments, *e.g.A*_*a*_^[*p*]^*B*_*b*_^[*q*]^*X*(1)_*x*1_^[*r*1]^*X*(2)_*x*2_^[*r*2]^. The rationalization and understanding of this coordinative segregation of the anions is one of the core aspects of the application of the CN rule. Mathematical application examples will be shown in Section 2.3. It has already been shown that Beck’s rule is able to correctly rationalize the coordination within certain structures, but no large-scale benchmark (as done for Pauling’s rules) has been performed (Beck, 2022 ▸; Voss & Kohlmann, 2017 ▸). This work therefore aims to automate the application of the extended CN rule and assess its rationalization power on a large dataset of crystal structures. The code to reproduce the results of this benchmark can be accessed via the linked repository (https://github.com/Niklas-Langer/ExtendedCoordinationNumberRule-Publication). Due to the fact that cations are only allowed to be coordinated by anions and vice versa, Beck’s rule is only applicable on ionic compounds. Therefore, a high difference in electronegativity in the investigated structures is desirable which lead to the selection of fluorides for this benchmark. In addition to being well suited for this type of investigation, fluorides have been described as model systems for structure–property relationships in ionic solids (Kraus, 2024 ▸). Since the solution of equation (1) is trivial when the cations show only a single CN, ternary compounds were chosen for investigation. Beck’s method has not yet been adapted for compounds containing unordered crystallographic sites (mixed positions or containing vacancies) as this would mean a mixture of local and global structural phenomena. Therefore, structures containing unordered sites were also omitted. The final dataset consisted of 2960 ternary fluorides as obtained from the Inorganic Crystal Structure Database of which 2161 did not feature unordered crystallographic sites (Zagorac *et al.*, 2019 ▸). Further restrictions caused by the algorithmic limitations will be explained in Section 3.5.

Finally, it should be noted that an anion-centered structural description can sometimes be advantageous for better illustrating basic structural building blocks or structure–property relationships (Krivovichev *et al.*, 2013 ▸). Of course, Beck’s rules can also be applied in this case by swapping the roles of cations and anions accordingly. In this article, however, we will retain the classic cation-centered view.

## 2. Theory

### 2.1. Describing the coordination in an ionic crystal structure

**Figure 1:**
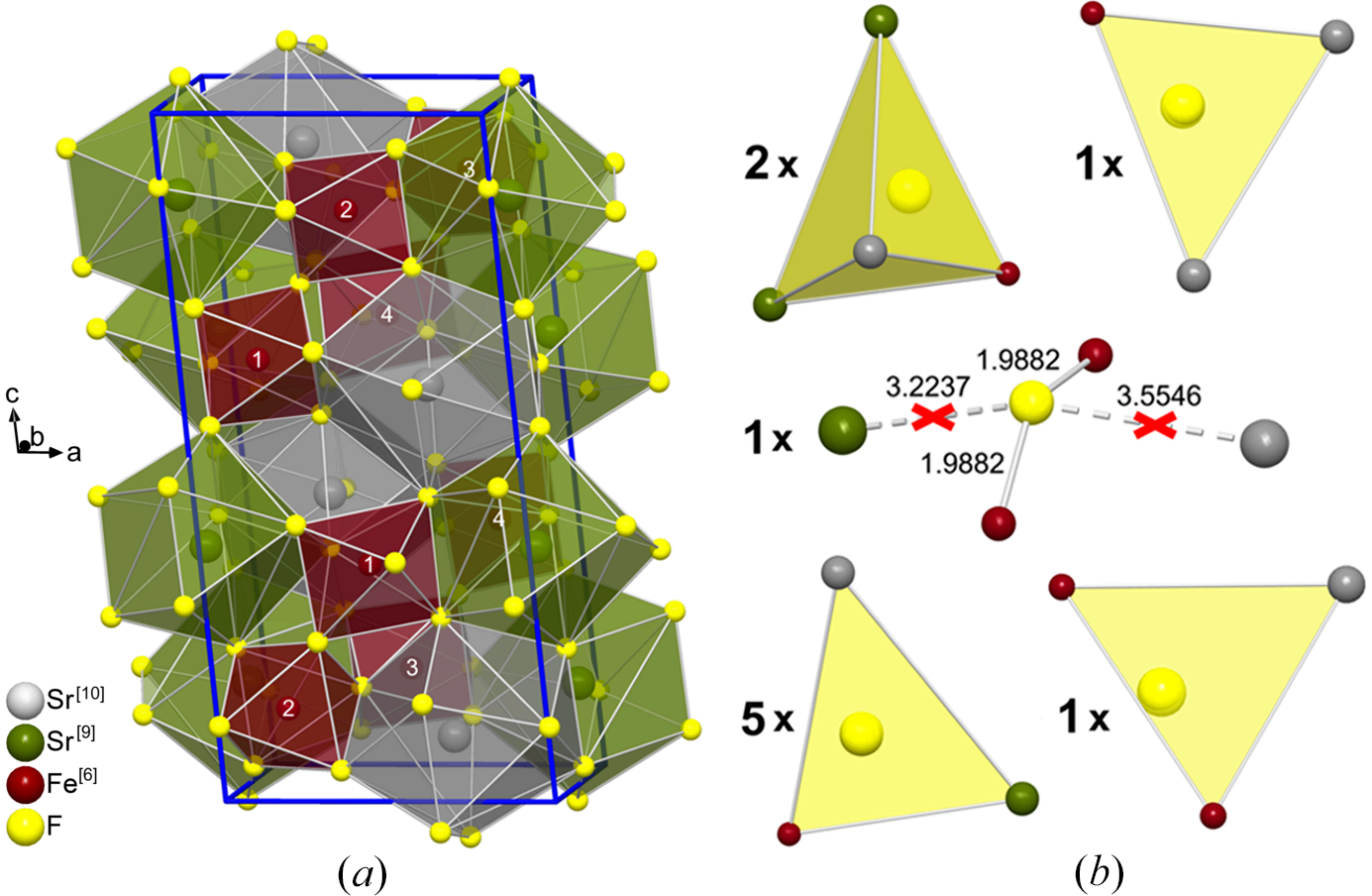
(*a*) Cation- and (*b*) anion-centered coordination polyhedra in SrFeF_5_. The numbers drawn as labels on the Fe^3+^ ions indicate in which way the helical chain is connected. The numbers shown next to the anionic coordination polyhedra indicate their respective counts within the doubled formula unit Sr^[10]^Sr^[9]^Fe_2_^[6]^F_10_. One fluoride anion shows a CN of 2 and is therefore shown with ionic bonds rather than a polyhedron.

To be able to rationalize the coordination within a structure using a fully automated approach, one must first translate the interatomic connectivity into a machine-readable format. This can be done using connectivity matrices (adjacency matrices) as known from theoretical chemistry. Since the CN rule can only be applied to ionic compounds in which the cations are only coordinated by anions and vice versa, half of the rows and columns can be omitted. This procedure will be shown on the crystal structure of SrFeF_5_ (Von der Mühll, 1973 ▸; Le Bail & Mercier, 1995 ▸). In this compound, the strontium atoms show two different CNs (9 and 10) as determined using the reciprocal largest gap method by Brunner implemented according to Section 3.2 (Brunner, 1977 ▸).^1^ These different CNs (indicated in square brackets) lead to a doubling of the formula unit to Sr^[10]^Sr^[9]^Fe_2_^[6]^F_10_. The coordinations within the structure can be shown either cation or anion centered, with the cation centered perspective being much more common in inorganic solid state chemistry. However, as shown in equation (1) both options are equally valid for the description of ionic compounds. The cation and anion centered polyhedra for SrFeF_5_ are shown in Fig. 1 ▸. Using a cation-centered perspective, the structure can be described as comprised of helical _∞_^1^{[FeF_4_F_2/2_]^2−^} chains built up from *cis*-connected corner-sharing [FeF_6_] octahedra. The unit cell contains two of such chains. The Fe^3+^ ions forming the chains alternately occupy two independent crystallographic sites (Le Bail & Mercier, 1995 ▸). Since both sites are coordinated octahedrally, they do not need to be distinguished in Beck’s theory. The [FeF_6_] octahedra share corners and edges with the [SrF_10_] and [SrF_9_] units, which themselves are also linked together by shared corners and edges. The [SrF_10_] polyhedra can be characterized as distorted bicapped square antiprisms whereas the [SrF_9_] polyhedra can be regarded as distorted monocapped square antiprisms (Le Bail & Mercier, 1995 ▸). A description using an anion-centered perspective is more complicated due to the lower CNs of the anions and the higher variety in their coordination environments. The structure is mainly comprised of trigonally planar coordinated F^−^ ions which are edge-connected and form ribbons. The fluoride anions in these ribbons are shifted out of the centers of their respective trigonal planes and are alternately moved slightly above and below the formed ribbon. The F^−^ ribbons are interconnected via corner- and edge-sharing with the tetrahedrally coordinated F^−^ ions and other trigonally planar coordinated fluoride anions. The F^−^ ions with CNs of 2 are also part of the interconnection of the ribbons.

**Figure 2:**
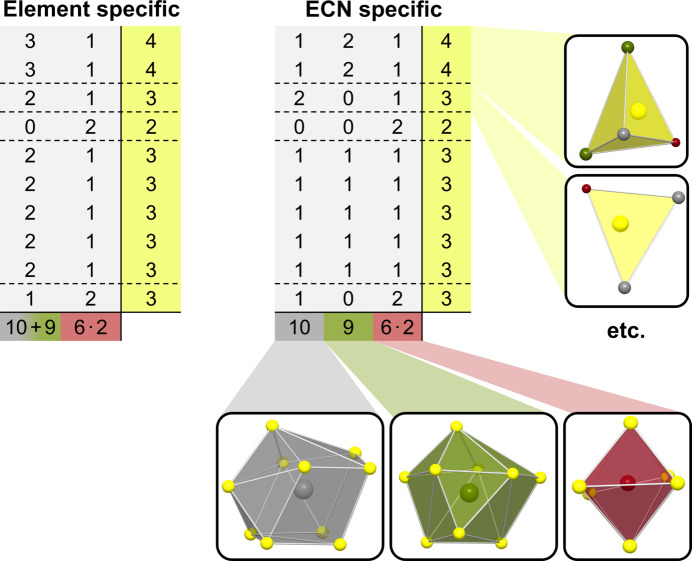
Element- and ECN-specific coordination matrices for the crystal structure of SrFeF_5_. In addition to the matrices, the sums of the rows (CNs of the anions) and the columns (CNs of the cations) as well as selected coordination environments represented by the notation are also shown.

The coordination of SrFeF_5_ can now be rationalized into a coordination matrix by assigning each of the cations a column and each of the anions a row within the matrix. For the crystal structure of SrFeF_5_ this results in the coordination matrix shown in Fig. 2 ▸. If one distinguishes between ions of the same species but with different CNs, the matrix will be called ECN specific (distinguished by element and CN). If only the chemical element is used for differentiation the matrix will be called element specific. The ECN-specific matrix can be converted into the element specific matrix simply by adding up the respective columns for one element. The element-specific matrix therefore contains less information.

### 2.2. Introduction of mathematical parameters

Assuming a ternary compound *A_a_*^[*p*]^*B_b_*^[*q*]^X*_x_*^[*r*]^ as described in Section 1, one can calculate the cationic contributions *r*_*A*_ and *r*_*B*_ to the CN *r* of the anion *X* using equation (1):
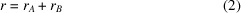
with
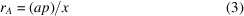
and
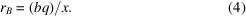


The parameters *r*_*A*_ and *r*_*B*_ are the average cation-specific CNs of the anion *X*. Beck’s extended CN rule is based on the assumption, that within an ionic structure *the cation-specific CNs of the anions tend to be as close to the average values r*_*A*_*and r*_*B*_*as possible to achieve a homogenous distribution of ions within the structure*. This deviation of the real coordination from the average cation-specific values is quantified as the *segregation index* Σ. It can be calculated using equation (5):
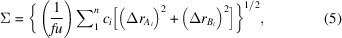
where *fu* is the number of formula units in the total formula, *c_i_* is the chemical index of equivalent anions (same coordination), *n* is the number of distinguishable anions (different coordinations) and the Δ*r* values give the deviations from the average cation-specific CNs *r*_*A*_ and *r*_*B*_. The coordination matrix for a given system that shows the most homogenous distributions of ions and therefore yields the lowest value for the segregation index Σ is called the simplest numerical solution (SNS). Beck also proposed a method how to calculate the SNS without calculating the segregation indices of all possible matrices. This method is called the principle of simplicity. It simply states that if any of the average CNs *r*, *r*_*A*_ and *r*_*B*_ are non-integer, the two adjacent integers are chosen instead.^2^ These restrictions only allow for the coordination matrices with the lowest segregation indices. The solution found using the *principle of simplicity* always shows a minimal segregation index and is therefore an SNS. An implementation of this mathematical procedure is explained in Section 3.4. In many cases the principle of simplicity is able to reduce the number of possible coordination matrices so much that only one solution remains. However, for more complicated matrices multiple matrices can be allowed, which can only be found using numerical methods. To calculate these matrices a brute force algorithm was implemented in addition to the calculation procedure proposed by Beck. The segregation index of the matrix with the lowest possible segregation index, the SNS, is called Σ_num_, whereas the segregation index of the coordination matrix of the experimentally determined structure is called Σ_tot_. If the coordination matrix of the experimentally determined structure deviates from the SNS this will lead to a higher value for the segregation index.^3^ This difference occurs due to chemical effects that stabilize the real structure with respect to the SNS and is therefore called Σ_chem_. It is defined as 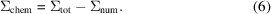


In many cases the difference Σ_chem_ can be explained by the charge of the ions which is not considered in the definition of the segregation index [equation (5)]. Thus, the most homogenous distributions of ions does not always represent the most homogenous distributions of charges. To account for that effect Beck proposed to sum over the deviations from the formal ion charges of the anions instead. The formal charges of the anions are calculated using the sums of the electrostatic bond strengths as proposed in Pauling’s second rule (electrostatic valence rule) (Pauling, 1929 ▸). This procedure yields a value which quantifies the local electroneutrality and which Beck therefore called the *electrostatic imbalance Y.* It is given by equation (7)
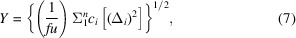
where the Δ values are the deviations from the formal charges of the anions. Similar to the segregation indices the electrostatic imbalance is called *Y*_num_ for the SNS and *Y*_tot_ for the experimentally determined structure. The difference of both values is defined as
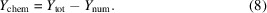


If the real structure shows a lower value for the electrostatic imbalance than the SNS, this will show as a negative value for *Y*_chem_. This indicates that the real structure exhibits a more homogenous distribution of charges than the SNS according to Pauling’s electrostatic valence rule. In many cases this finding can be used as an explanation of why the experimentally determined structure is preferred to the SNS. This combination of both concepts, the segregation index Σ and the electrostatic imbalance *Y*, gave rise to the name *extended coordination number rule*.

### 2.3. Application examples of Beck’s extended CN rule

The application of Beck’s CN rule will be shown exemplary on the structures of K_2_NiF_4_ and SrFeF_5_, whose structure was already presented in Section 2.1.

#### 2.3.1. Application example 1: K_2_NiF_4_

**Figure 3:**
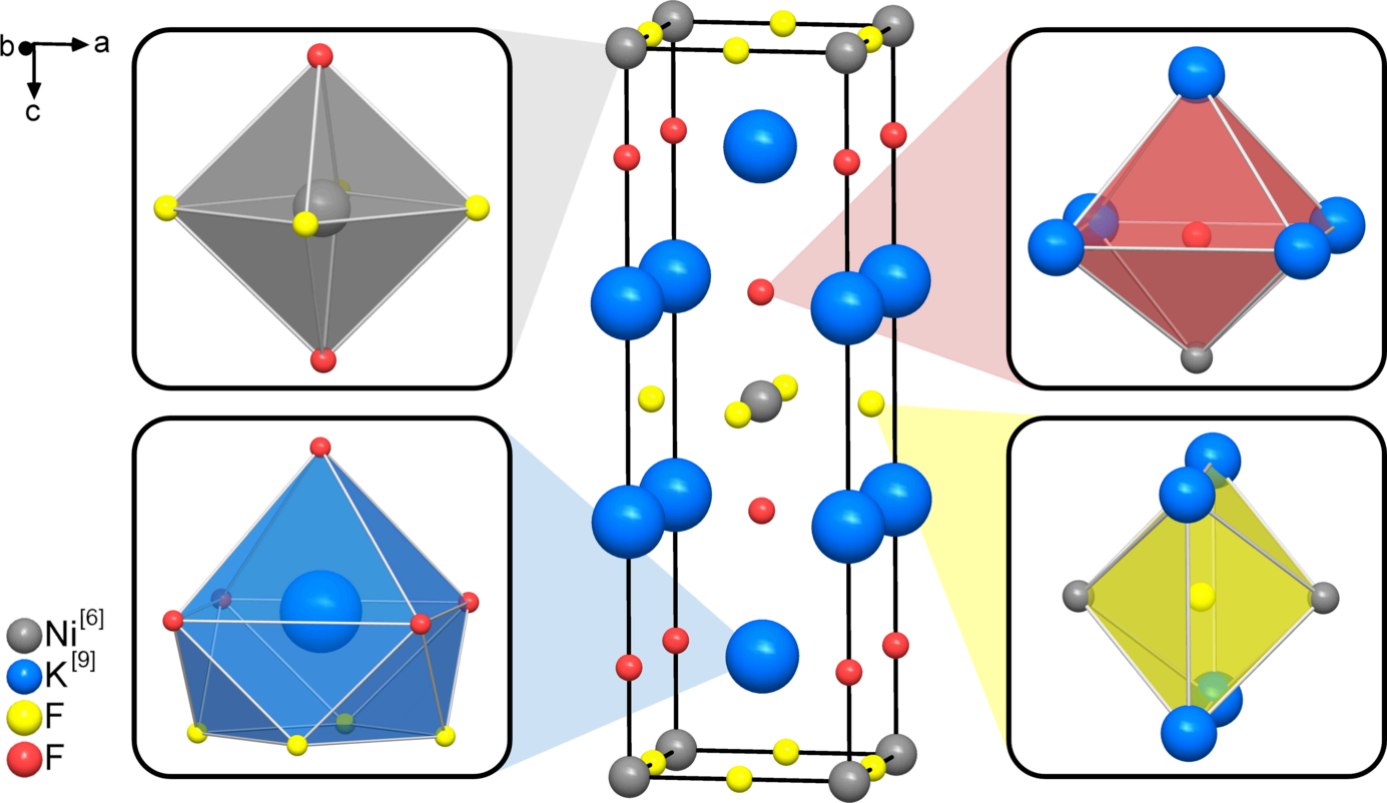
Crystal structure of K_2_NiF_4_ with cation- and anion-centered polyhedra.

K_2_NiF_4_ is the simplest entry (*n* = 1) to the structural group of the Ruddlesden–Popper phases. In the crystal structure of K_2_NiF_4_ the Ni^2+^ ions are coordinated octahedrally by six F^−^ anions, whereas the K^+^ ions are coordinated by nine F^−^ anions each. The crystal structure with the coordination environments for cations and anions is shown in Fig. 3 ▸. With the CNs indicated in square brackets the formula can be written as K_2_^[9]^Ni^[6]^F_4_. According to equation (1) the average CN of the fluoride anions can be calculated as *r* = (2 × 9 + 6)/4 = 6. According to the extended CN rule, all anions should therefore display a CN of 6. This is in agreement with the experimentally determined structure as shown in Fig. 3 ▸.

**Figure 4:**
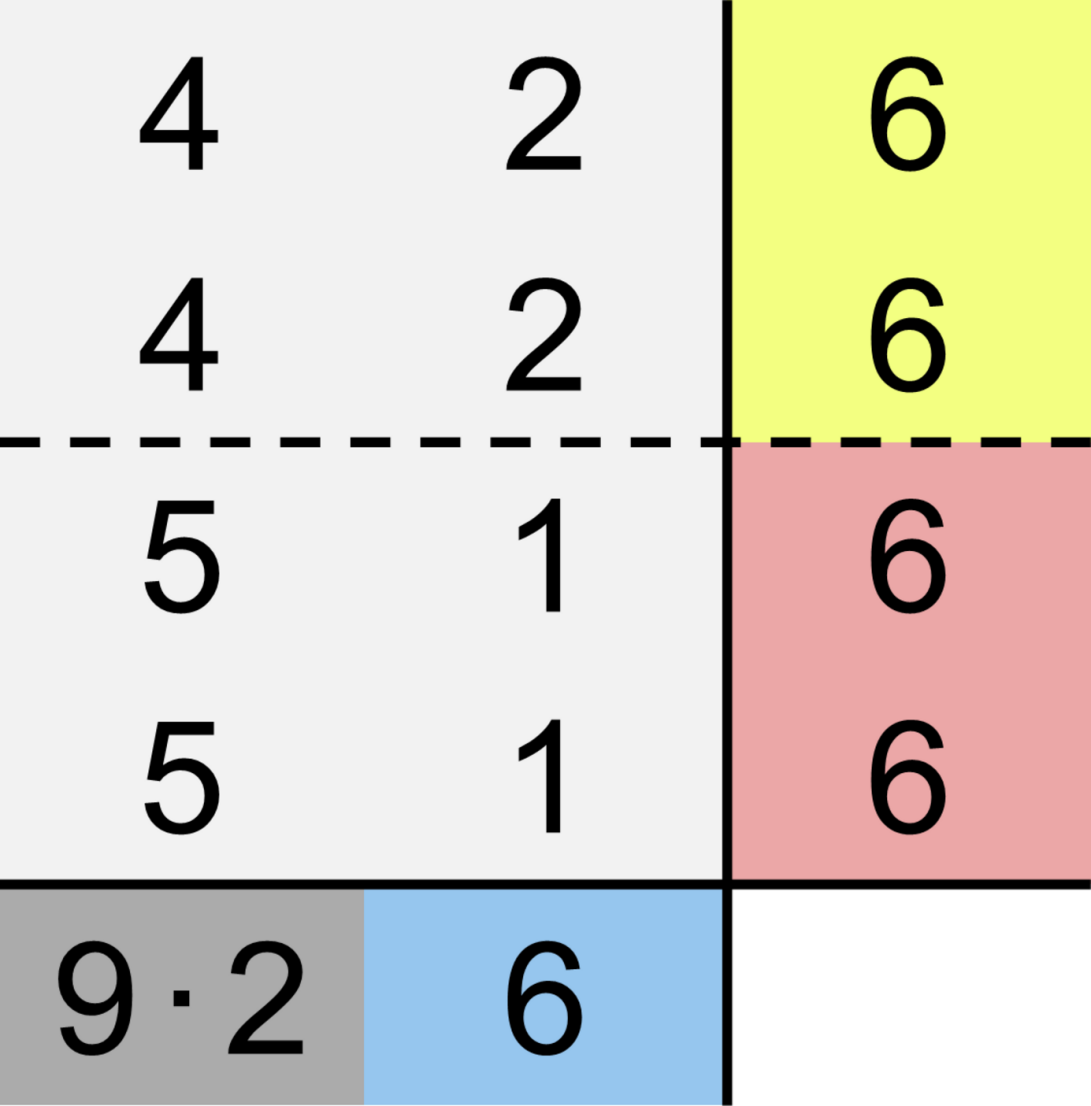
Coordination matrix for the SNS and experimentally determined structure of K_2_NiF_4_. The column and row sums are color coded according to the colors of the atoms shown in Fig. 3 ▸.

The next step in the application of Beck’s rule is to calculate the contributions *r*_K_ and *r*_Ni_ of the different cations to the anion coordination. This can be achieved using equations (3) and (4), which yield *r*_K_ = (2 × 9)/4 = 4.5 and *r*_Ni_ = 6/4 = 1.5. Chemically it does not make a lot of sense that one F^−^ ion should coordinate to 4.5 K^+^ ions and 1.5 Ni^2+^ ions. According to the *principle of simplicity* the two adjacent integers are chosen instead. This means that half of the anions are supposed to coordinate to four K^+^ ions each whereas the other half is expected to coordinate to five K^+^ ions each. Accordingly, half of the anions are supposed to coordinate to one Ni^2+^ ion whereas the other half is expected to coordinate to two Ni^2+^ ions each. Since the CNs of the anions (as sums of the cationic contributions *r*_K_ and *r*_Ni_) are already calculated to be 6, only one coordination matrix is possible according to Beck’s extended CN rule which is shown in Fig. 4 ▸. This coordination matrix, the SNS, is in complete agreement with the coordination environments in the experimentally determined structure as shown in Fig. 3 ▸. The restrictions obtained from the calculation of *r*, *r*_K_ and *r*_Ni_ (*principle of simplicity*) only allow this single coordination matrix.

#### 2.3.2. Application example 2: SrFeF_5_

The coordination environments within the crystal structure of SrFeF_5_ have been readily described in Section 2.1. To include the CNs its chemical formula can be written as Sr^[10]^Sr^[9]^Fe_2_^[6]^F_10_. According to equation (1) the average CN of the anions is calculated as *r* = (10 + 9 + 2 × 6)/10 = 3.1. As stated by the *principle of simplicity* the adjacent integers of 3 and 4 are chosen instead. This means that nine out of ten F^−^ anions are supposed to show a CN of 3 whereas one F^−^ anion is expected to display a CN of 4. The contributions of the different cations are now calculated ECN specifically, which means that the Sr^2+^ ions with the different CNs are kept separated. According to equation (3) the following values are obtained: *r*_Sr[10]_ = 10/10 = 1, *r*_Sr[9]_ = 9/10 = 0.9 and *r*_Fe_ = (2 × 6)/10 = 1.2. For *r*_Sr[9]_ and *r*_Fe_ non integer values are obtained, thus the adjacent integers will be used instead. In combination with the CNs of the anions as calculated from *r* a total of 4 restrictions has been obtained from the *principle of simplicity*:

(1) Nine out of ten F^−^ anions show a CN of 3, whereas one F^−^ anion displays CN 4.

(2) All ten F^−^ anions coordinate to one tenfold coordinated Sr^2+^ ion each.

(3) Nine out of ten F^−^ anions coordinate to one ninefold coordinated Sr^2+^ ion each, whereas the other F^−^ anion does not coordinate to a ninefold coordinated Sr^2+^ ion.

(4) Eight out of ten F^−^ anions coordinate to one Fe^3+^ ion, whereas the other two F^−^ anions coordinate to two Fe^3+^ ions each.

**Figure 5:**
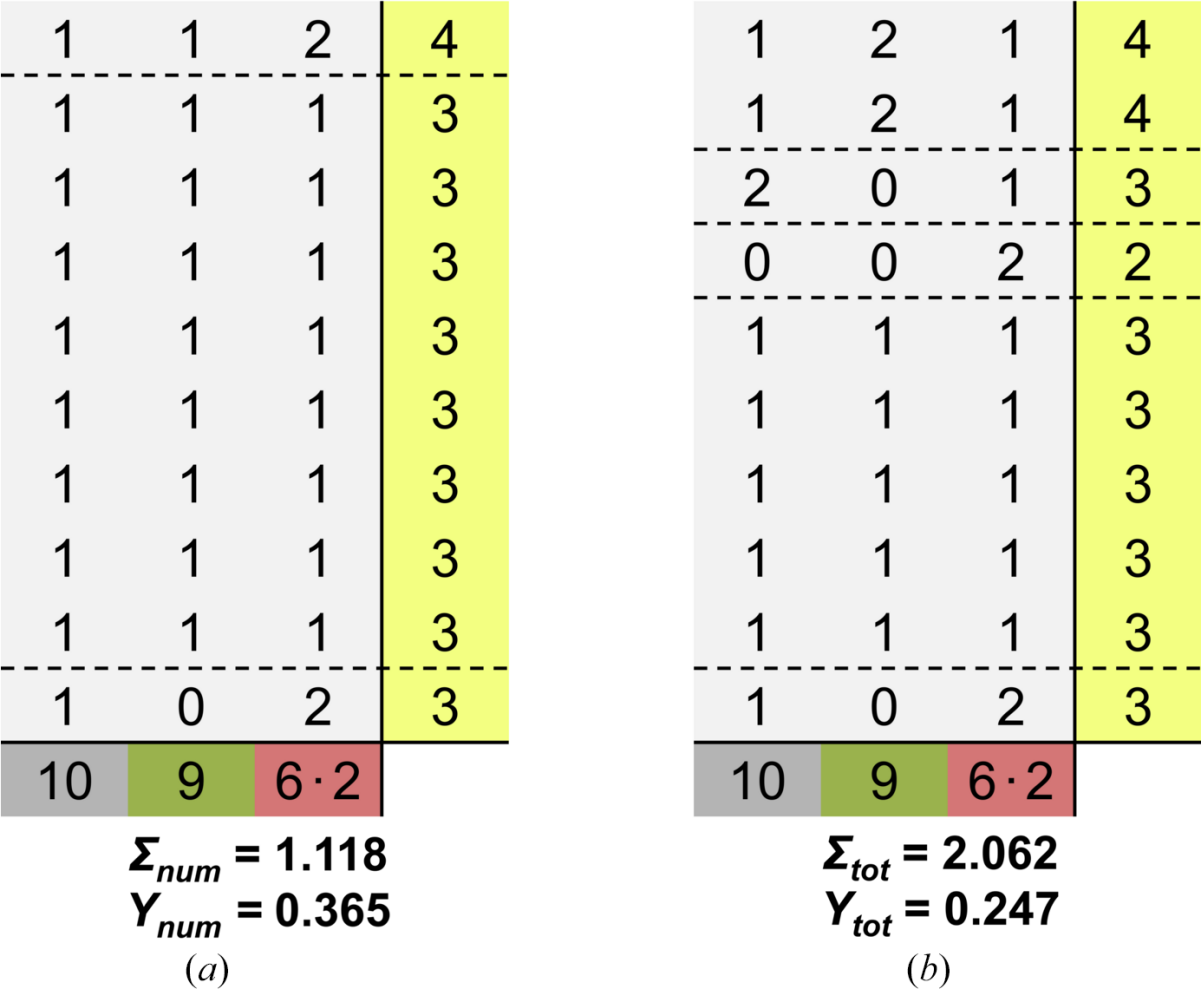
Coordination matrices of (*a*) SNS and (*b*) the experimentally determined structure of SrFeF_5_ with the respective segregation indices Σ_num_ and Σ_tot_ and electrostatic imbalances *Y*_num_ and *Y*_tot_.

Similar to the first example, only one coordination matrix fulfills all four restrictions. It is shown in Fig. 5 ▸(*a*) in comparison to the coordination matrix of the experimentally determined structure (right). The SNS shows a significantly lower segregation index of 1.118 than the experimentally determined structure with 2.062. This deviation can be explained by calculating the electrostatic imbalance *Y*, which assumes a value of 0.365 for the SNS and a much lower value of 0.247 for the experimentally determined structure. The reason for this difference can be found by comparing the contributions of each row. Especially the first row of the SNS matrix shows a very high deviation from the charge of the fluoride anions of −1. The negative sum of the electrostatic bond strengths of the first row yields a value of −(2/10 + 2/9 + 2 × 3/6) = −1.42. The experimentally determined structure assumes a more complicated coordination to avoid this. The main difference to the SNS is the fourth row, which only shows a CN of 2. By only coordinating to two Fe^3+^ ions this anion achieves local charge neutrality by yielding a value of −1.0 for the negative sum of electrostatic bond strengths.

As mentioned in Section 2.1 some authors suggest CNs of 9 for all strontium ions present in the structure, thus yielding the formula Sr^[9]^Fe^[6]^F_5_. Repeating the calculation with this new input also yields a disagreement between the SNS and the experimentally determined structure. The segregation indices and the electrostatic imbalances show values of Σ_num_ = 1.265 and *Y*_num_ = 0.248 for the SNS and Σ_tot_ = 1.897 and *Y*_tot_ = 0.222 for the experimentally determined structure. The structural deviation from the SNS can therefore be explained by a lower electrostatic imbalance. This result is the same as for the original calculation with CNs of 9 and 10 for the strontium ions. Choosing coordination of only 10 for the strontium ions (Sr^[10]^Fe^[6]^F_5_) also yields similar results with values of Σ_num_ = 0.894 and *Y*_num_ = 0.447 and Σ_tot_ = 1.673 and *Y*_tot_ = 0.283.

## 3. Implementation

### 3.1. Overview of software architecture

**Figure 6:**
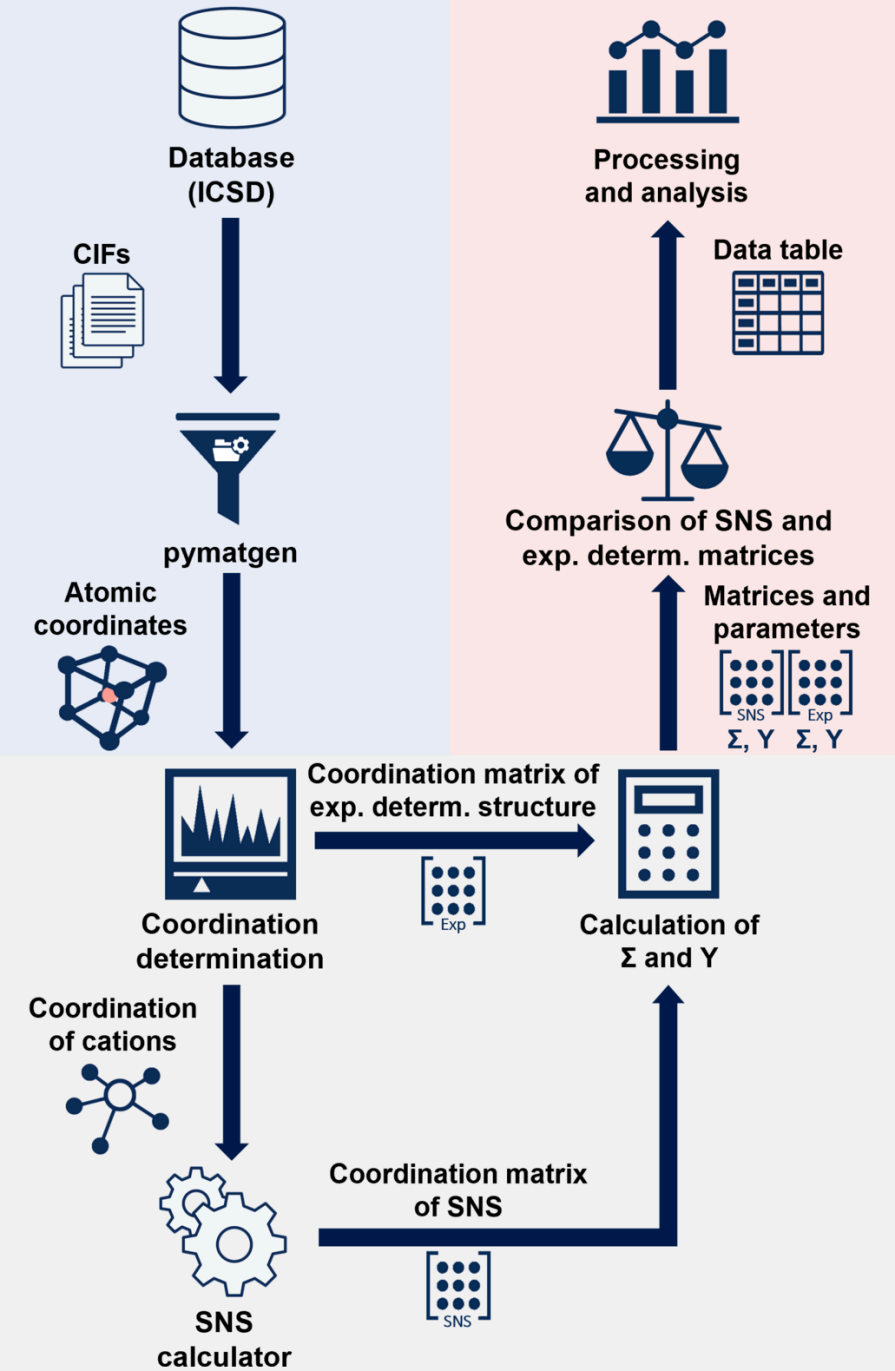
Schematic overview of the procedure which the developed algorithm follows. The three main parts of the program are shown in different colors: import (blue), coordination analysis (gray) and data output (red).

The developed algorithm follows a procedure, of which a simplified version is shown in Fig. 6 ▸. Except for gathering of the CIFs from the database (which is done in a separate process using the ICSD API), all steps are integrated into a single program which can process any number of given CIFs fully automatically.

The CIFs of the ternary fluorides were gathered using the ICSD API (Zagorac *et al.*, 2019 ▸). The atomic coordinates are generated from the CIFs using the pymatgen library (Jain *et al.*, 2013 ▸). In the next step the coordination environments of all atoms are determined, and a coordination matrix is generated. The program was tested with different methods for the CN determination which will be described in Section 3.2. Afterwards the CNs of the cations and the chemical index of the anions are used to calculate the SNS. For both, the coordination matrix of the experimentally determined structure and the SNS, the segregation index and the electrostatic imbalance are calculated. Then both matrices as well as their mathematical parameters are compared, and the results are written to a tabular output file.

### 3.2. CN determination

A multitude of methods for CN determination are described in the literature. The ones that were considered and tested for the treatment of the dataset are the normal and reciprocal largest gap method as described by Brunner (1977 ▸), the ECoN method proposed by Hoppe (1979 ▸), the ChemEnv tool available in the pymatgen library which performs a Voronoï analysis similar to the approach by O’Keefe (Waroquiers *et al.*, 2020 ▸; O’Keeffe, 1979 ▸) and the CrystalNN algorithm (Pan *et al.*, 2021 ▸). Results for all methods can be reproduced using the code available in the repository. The method that was ultimately chosen for further analysis was the reciprocal largest gap method by Brunner, which searches for the largest gap in the histogram of reciprocal interatomic distances. The main reason for its selection was that due to its simplicity it could be easily modified to fit the needs of the dataset consisting of ternary fluorides while also not violating equation (1).^4^ It is also the method that Beck used most frequently with his theory (Beck, 2014 ▸). Since the prerequisite for the application of the extended CN rule is an equality of the sums of cationic and anionic CNs, the anionic coordination has to match the cationic coordination. Covalent interactions between two cations or two anions are also not allowed as they would violate this principle. This is achieved by only determining the coordination environments for the cations and applying these back to the anions and only considering coordination between ions of different charge signs. Due to the non-directional nature of the ionic bonding present in fluorides, cationic CNs smaller than 3 are unlikely to appear. CNs higher than 12 are also unlikely as they are usually only featured in intermetallics. These properties are implemented by restricting the search for the largest gap to 3 ≤ CN ≤ 12. Another feature of ionic structures is that interactions of ions with the same charge are highly unfavorable. While the imposed restrictions already permit that two cations coordinate each other, it is still possible that a cation is present within the coordination sphere of another cation while not being considered coordinated. This is prevented by inspecting the interatomic distances of all surrounding ions and not just for the anions. If for a certain CN another cation is closer to the central cation than an anion within its coordination sphere, this CN is not considered, and a smaller number of neighbors is chosen instead.

### 3.3. Charge determination

For the calculation of the electrostatic imbalance the formal charges of all ions present in the structure must be known. All CIFs obtained from the ICSD come with predetermined oxidation states. To assure their correctness they were checked using a simple algorithm developed by one of the authors which assigns the formal charges based on the common oxidation states for each element as described by Greenwood & Earnshaw (1997 ▸). Another correctness check was performed using the BERTOS algorithm (Fu *et al.*, 2023 ▸). If the methods yielded different results, the oxidation states were checked manually by consulting the respective publication. This was the case for 114 out of 2161 ternary fluorides. The main reasons for the disagreement were the featuring of covalent bonds or atoms of the same chemical element showing different oxidation states within the same structure (*e.g.* CsFe_2_F_6_).

### 3.4. Solving algorithm

**Figure 7:**
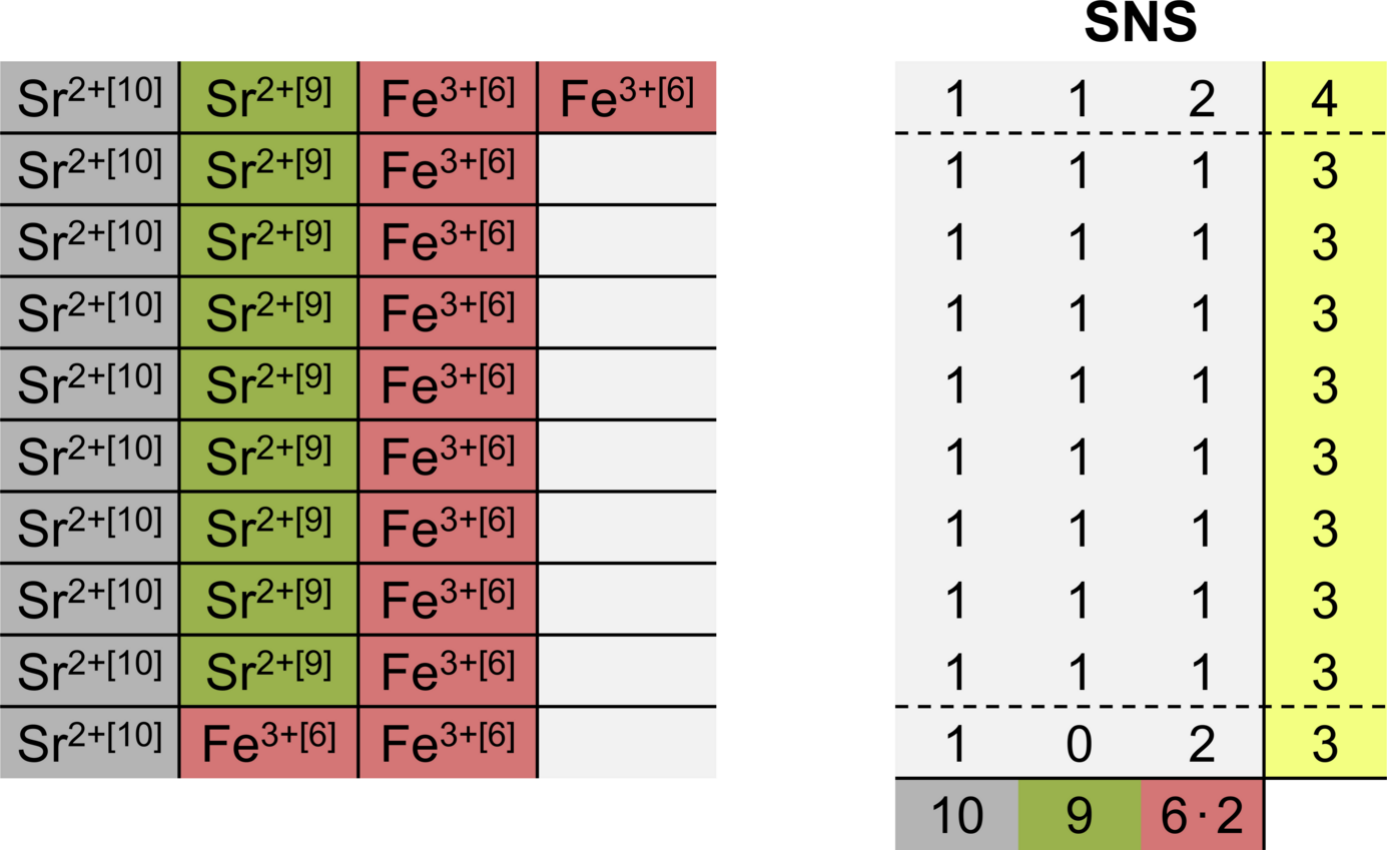
Determination of the SNS using the marble method. By inserting the cation-anion-contacts one ECN site after each other, the most homogenous distribution of cation–anion contacts and therefore the minimal segregation index is achieved.

The application of the *principle of simplicity* yields a set of restrictions which the SNS needs to obey. There are multiple ways to calculate an SNS. The easiest option (proposed by Beck himself) is to imagine each cation–anion contact as a marble which rolls into a certain number of slots. The number of cation–anion contacts is given by the CN of each cation, and the number of slots is given by the chemical index of the anions. Each ECN site has its own set of ‘marbles’ which one can imagine being in different colors. If the ‘marbles’ (representing the cation–anion contacts) are inserted one color (ECN site) after each other, this always leads to the most homogenous distribution of cation–anion contacts and therefore a minimal segregation index. This is illustrated in Fig. 7 ▸ on the example of SrFeF_5_ as described in Section 2.3.2.

For most structures the order in which the different colors are inserted does not play a role and all orderings result in the same SNS matrix. However, for certain structures (especially more complicated structures featuring high numbers of ECN sites) it is possible that more than one SNS exists. This means that the order in which the cation–anion-contacts are inserted will result in different matrices. However, the ‘marble method’ is only able to determine as many SNS matrices, as the possible permutations of the ECN sites (*e.g.* three ECN sites will result in a maximum of six different permutations/SNS). For certain structures even more SNS exist. These correspond to matrices in which the marbles are not inserted one color after each other but in a mixed way or leaving holes. To find these SNS a brute force algorithm was implemented, that calculates the segregation indices for all possible matrices. Brute force calculations were carried out for 94% of the crystal structures contained in the final dataset. For the other structures calculation times on the available computational resources were too high. All SNS determined using the simple marble algorithm agreed with the matrices calculated using the brute force algorithm. Around 7% of the structures showed more than one SNS. It could be shown that in rare cases coordination matrices can exist that show the same minimal segregation index as the SNS despite not obeying the *principle of simplicity* (see Section 4.1).

### 3.5. Overview of the dataset

The dataset on which the analysis was performed was generated using all 2960 ternary fluorides listed in the ICSD as of the 2025.1 release (Zagorac *et al.*, 2019 ▸). Crystal structures containing unordered sites (mixed positions or positions containing vacancies) were excluded, leaving 2161 entries. [This was done because the concept *extended coordination rule* is based on local order, whereas unordered sites are a global phenomenon. Since compounds containing unordered sites do not generally behave differently than their counterparts, which only contain fully occupied sites, this should not have a negative impact on the validity of the presented results. Future investigations will still aim to broaden Beck’s theory to include these compounds.] Structures in which ions of the same element show different charges were also omitted for reasons of simplicity. This leaves 2114 crystal structures. Compounds containing multiple anions (*e.g.* FeOF) are also excluded but can be included in future investigations by inverting cationic and anionic input. However, this has not been mentioned in Beck’s original publications and is, therefore, not part of this work. Also excluded were compounds consisting exclusively of non-metals and crystal structures of Xe and Kr compounds due to their molecular nature. This leaves 1649 compounds. In the last data reduction step, it was checked whether there are compounds in which anions show a CN of 0. For cations these checks do not need to be performed since this is actively prevented by the CN determination algorithm as described in Section 3.2. In ten compounds anions were assigned a CN of 0. This is obviously not correct. A manual check revealed that this issue was mostly based on complex anions being present in the structures. It would still be possible to manually assign the CNs in a way that prevents values of 0, but that would make the acquired data inconsistent and would also disagree with some of the structural descriptions made in the original publications. These crystal structures were therefore omitted. This leaves a total of 1633 remaining structures in the dataset. For six structures, errors were found in the deposited datasets which were therefore excluded.^5^The ICSD often contains duplicate entries for the same crystal structure. This could generate a bias in the generated data which makes a duplicate removal necessary. This is done by checking whether multiple entries show the same chemical formula as well as the same cationic CNs. If that is the case only the first entry is kept. The final dataset after the duplicate removal contained 1038 crystal structures.

## 4. Results and discussion

### 4.1. General rationalization power

By applying the developed algorithm to the dataset consisting of 1038 ternary fluorides the rationalization power of the extended CN rule was examined. The term ‘rationalization power’ will be used in this publication to indicate whether a compound fulfills Beck’s extended CN rule. This means that either there is an agreement between the SNS and the coordination matrix of the experimentally determined structure or that the deviation can be explained by a lower electrostatic imbalance. The term ‘agreement’ will be used only if the SNS and experimentally determined coordination matrix are equal. The results are summarized in Table 1 ▸. It could be shown that there is an agreement of the ECN-specific coordination for 59.9% of the crystal structures. When columns belonging to the same element but with different CNs are summed together to generate an element-specific matrix, the agreement rises to 62.9%. In 22.1% of the crystal structures there is a disagreement between the SNS and the coordination matrix of the experimentally determined structure, which can be explained by a lower value of the electrostatic imbalance (*Y*_tot_ < *Y*_num_). When the equality of *Y*_tot_ and *Y*_num_ is also considered explainable, the value rises to 22.3%. In total, 82.2% of the examined crystal structures either agree with the SNS or can at least be explained electrostatically on an ECN-specific basis using Beck’s extended CN rule. Furthermore, for 24 structures (2.3%) the coordination matrices of the experimentally determined structures show a minimal segregation index while not obeying the *principle of simplicity*. Those matrices are not considered simplest solutions and the structures therefore are not regarded as agreeing with the SNS. For only 1 of these 24 structures the deviation from the SNS was explainable using the electrostatic imbalance.

### 4.2. Influence factors on the rationalization power

#### 4.2.1. Influence of structural complexity

**Figure 8:**
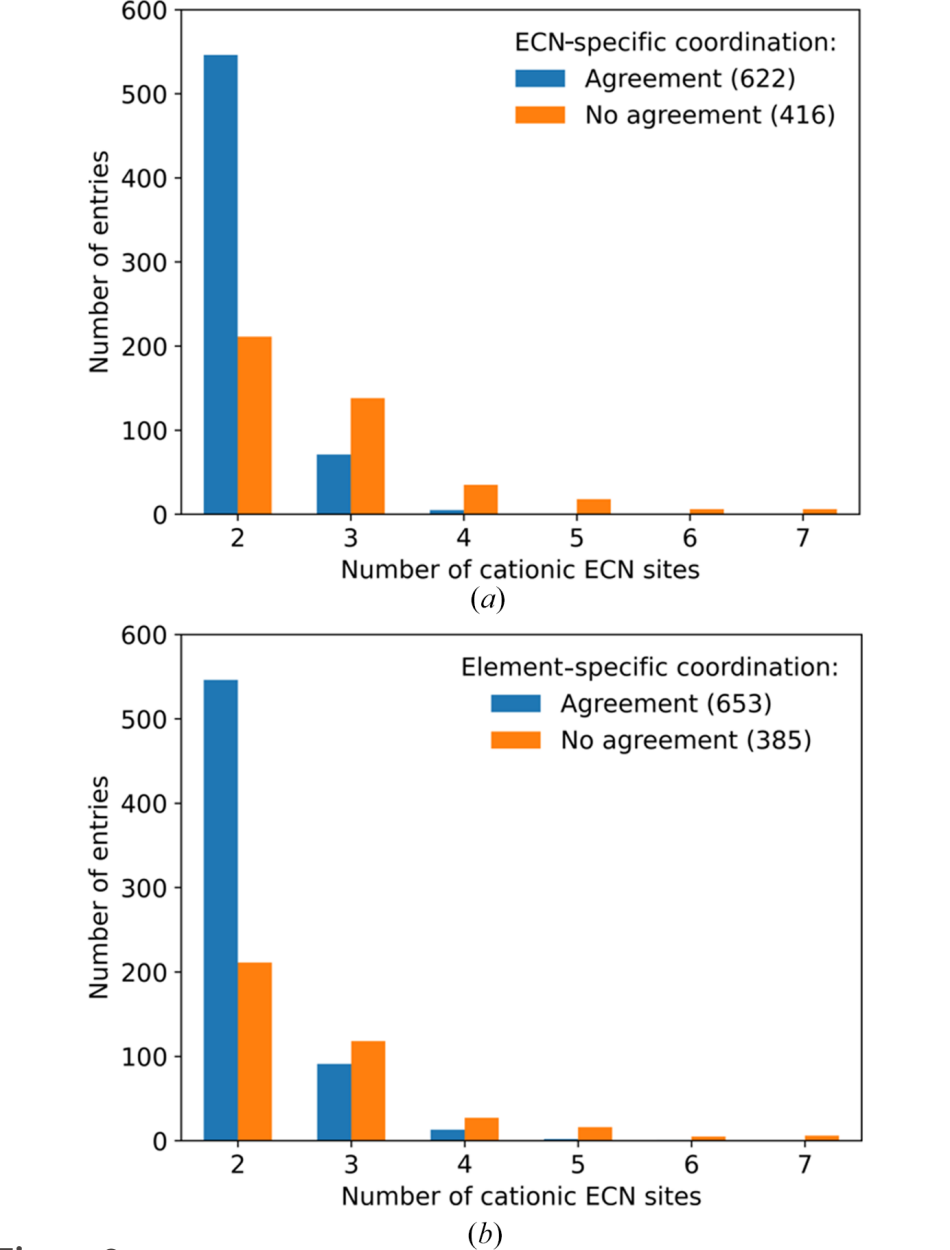
Agreement between (*a*) ECN- and (*b*) element-specific coordination matrices of the SNS and the experimentally determined structure in subdatasets with different numbers of cationic ECN sites.

The number of ECN sites present within a compound determines the number of columns of the respective coordination matrix. If a matrix has more columns it allows for more possible permutations of the cation–anion contacts. This means more allowed matrices exist and the differences in their segregation indices are lower than for matrices with fewer columns. Based on this assumption the rationalization power of the extended CN rule is expected to decrease for structures containing higher number of cationic ECN sites. This behavior could be observed and is shown in Fig. 8 ▸. It is clearly visible that a higher number of cationic ECN sites leads to a lower agreement between the coordination in the experimentally determined structure and the SNS. Only for compounds containing two cationic ECN sites there is a reasonable agreement which reaches a value of 72%. However, due to the fact that in most ternary fluorides only a single ECN site is featured per cationic species, the group containing only two cationic ECN sites is by far the biggest and the high agreement here outweighs the lower agreement for higher numbers of ECN sites. This preference of compounds to feature low numbers of distinguishable ECN sites can be linked to Pauling’s rule of parsimony. For compounds containing three or more ECN sites the agreement rapidly drops below 50%. This behavior is less pronounced for the element-specific matrices since the columns for each cation are added together. This makes structures featuring three ECN sites more similar to compounds containing only two ECN sites. The observation that the agreement is still below 50% might be explainable by the way the element-specific matrices are created. At the moment the element-specific SNS is treated as a simplification of the ECN-specific matrix. In future studies, it might be better to carry out the ECN and element-specific calculations separately since for the element-specific matrix is less restricted by the *principle of simplicity*.

**Figure 9:**
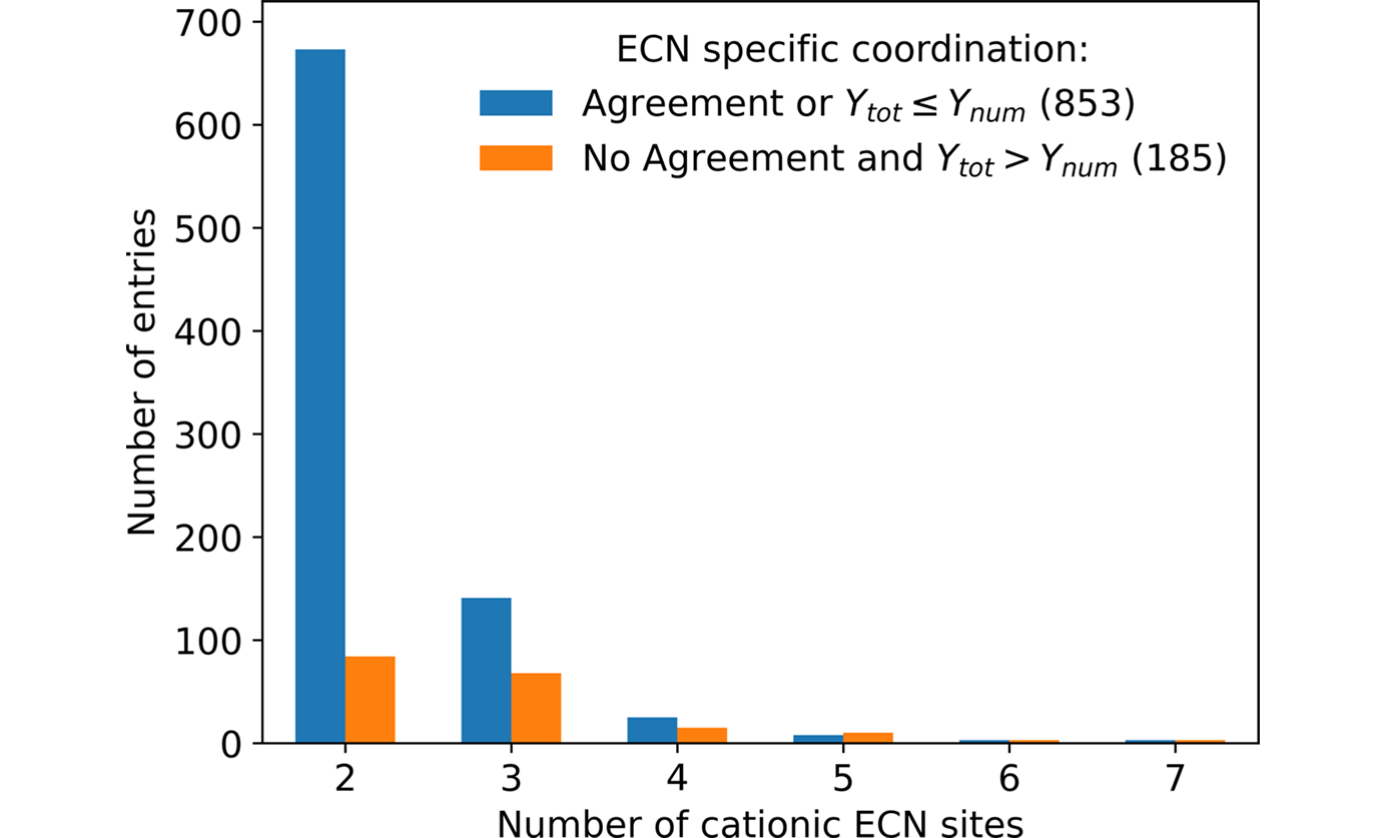
Rationalization power (including agreement between SNS and experimentally determined structure as well as explainability via electrostatic imbalance) of Beck’s extended CN rule in subdatasets with different numbers of cationic ECN sites.

The situation changes when the matrices that can be explained by the electrostatic imbalance *Y*_tot_ being less or equal to *Y*_num_ are also included on the agreement side as it is shown in Fig. 9 ▸. While the number of agreeing/explainable structures still drops with increasing numbers of cationic ECN sites, there is still a reasonable rationalization power for structures containing up to four different cationic ECN sites.

#### 4.2.2. Influence of the chemical composition

**Figure 10:**
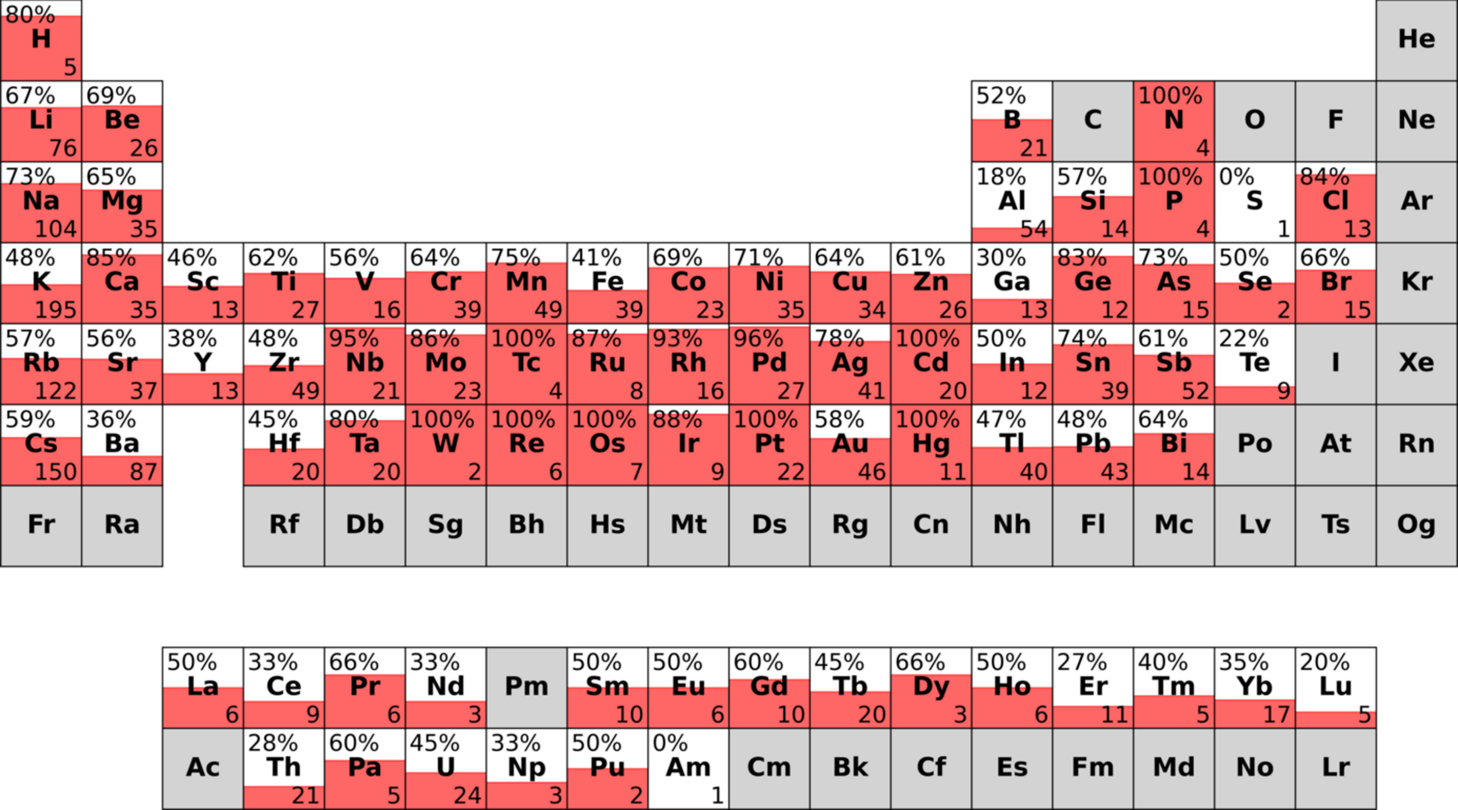
Agreement between ECN-specific coordination matrices of the SNS and the experimentally determined structure in subdatasets for each chemical element. The number on the top left of each element indicates the agreement which is also represented by the filling. The number on the bottom right corresponds to the size of the subdataset for the respective chemical elements. Elements for which no data was available are colored gray.

The properties of a compound are determined by its chemical composition and its crystal structure. It is therefore expected that certain classes of substances show different agreement with the extended CN rule. This was examined by dividing the generated dataset into subdatasets for each chemical element of the cations. For example, the compound SrFeF_5_ would be added to the Sr and Fe subdatasets. Plotting the ECN-specific agreement for each subdataset yields the plot shown in Fig. 10 ▸.

**Figure 11:**
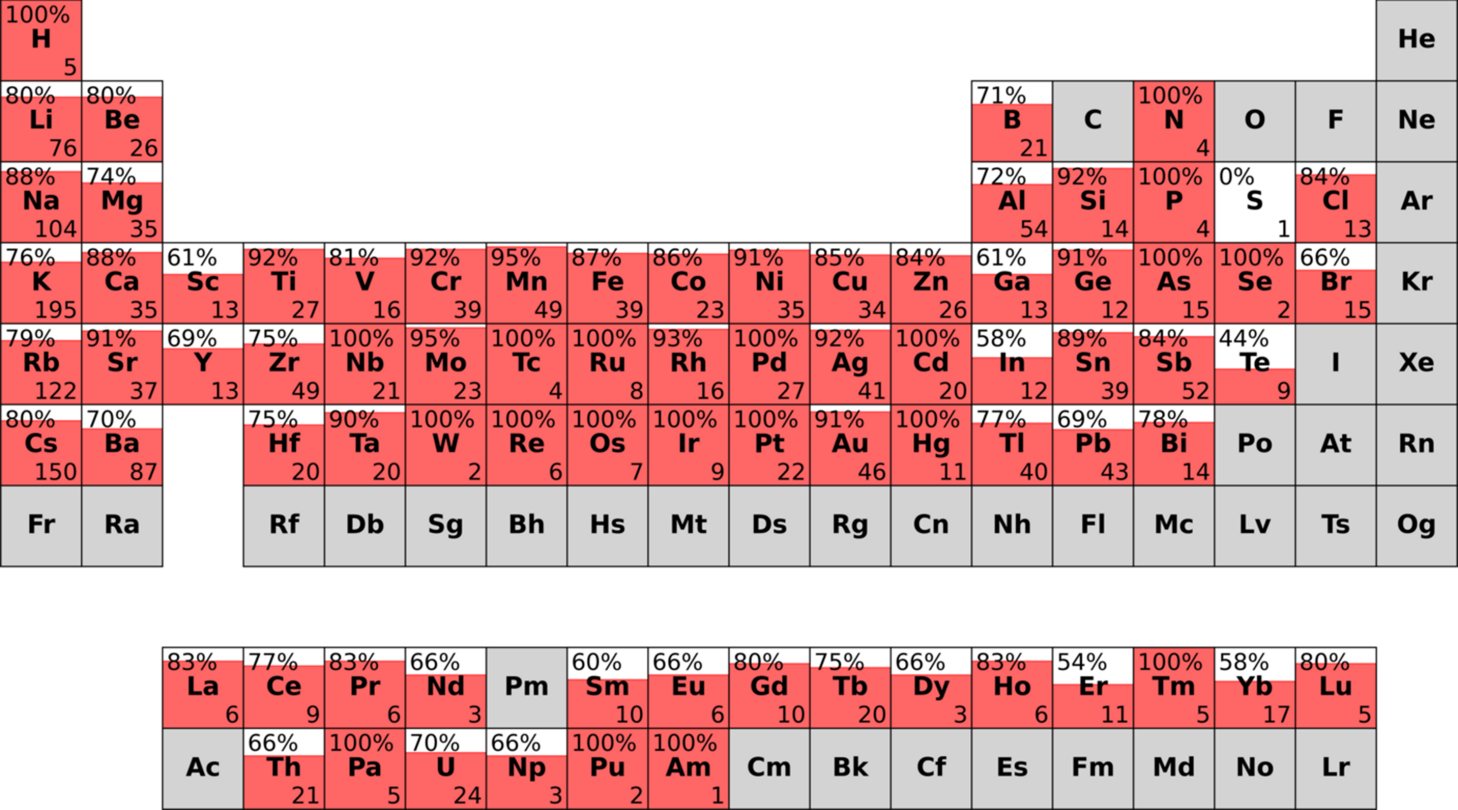
Rationalization power (including agreement between SNS and experimentally determined structure as well as explainability via electrostatic imbalance) of Beck’s extended CN rule in subdatasets for each chemical element. The number on the top left of each element indicates the agreement which is also represented by the filling. The number on the bottom right corresponds to the size of the subdataset for the respective chemical elements. Elements for which no data was available are colored gray.

Fig. 10 ▸ shows a high agreement across most parts of the periodic table with the main exception being the group 3, 13 and 16 elements as well as the lanthanides and actinides. For the group 3, 13 and *f*-block elements this lower agreement is most likely originating from the relatively high charges these elements assume. This makes it likely that this disagreement between SNS and experimentally determined structure is explainable by the electrostatic imbalance. Including the *Y*_tot_ ≤ *Y*_num_ subdataset on the agreement side yields the plot shown in Fig. 11 ▸.

**Figure 12:**
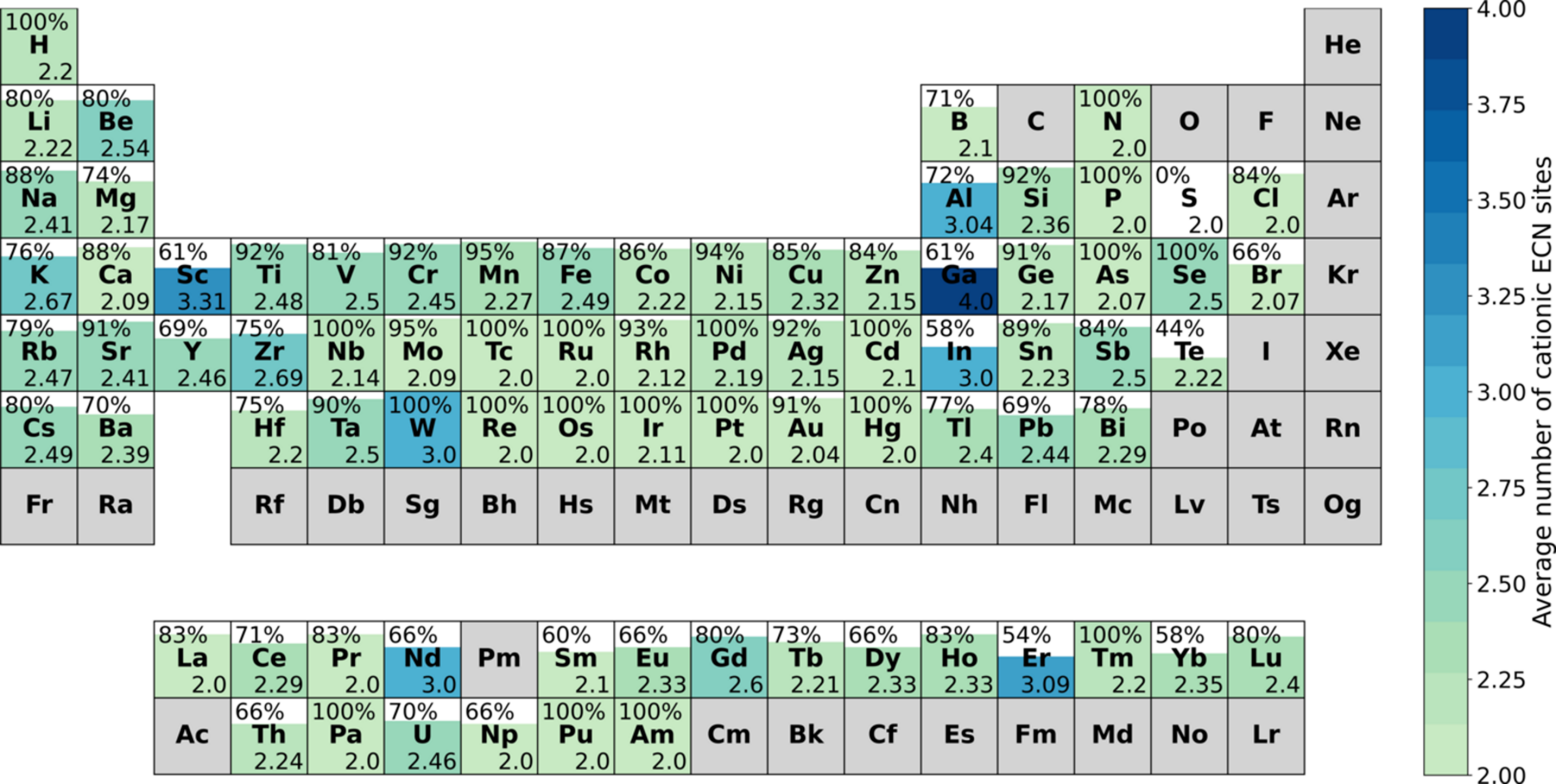
Rationalization power (including agreement between SNS and experimentally determined structure as well as explainability via electrostatic imbalance) of Beck’s extended CN rule in subdatasets for each chemical element. The number on the top left of each element indicates the agreement which is also represented by the filling. The number on the bottom right corresponds average number of cationic ECN sites in the subdataset for the respective chemical element. This value is also expressed via the color gradient. Elements for which no data was available are colored gray.

It can clearly be seen that especially aluminium and gallium containing compounds can be rationalized overproportionally well when the electrostatic imbalance is included. The average rise for each element is equal to the relative size of the *Y*_tot_ ≤ *Y*_num_ subdataset with 22.3%. However, the observed increase for Al and Ga compounds is much higher, which indicates an overproportional rationalization power based on the electrostatic imbalance rather than the segregation index. This result could be related to the analysis published by George, which suggests that oxide anions surrounded by aluminium ions tend to fulfill Pauling’s second rule best. However, since George’s investigation was conducted on oxides it remains unclear whether this finding can be transferred to fluorides. For the other elements mentioned to show a high fulfillment of Pauling’s second rule (Si, Sn, Sc, Rh, Pd, Ir) this effect is less pronounced (George *et al.*, 2020 ▸). This might be due to the already relatively good agreement even without the inclusion of the *Y*_tot_ ≤ *Y*_num_ subdataset (Fig. 11 ▸). Building on the results described in Section 4.2.1 one can now also include the average number of cationic ECN sites for each chemical element in the plot (Fig. 12 ▸). For nearly all chemical elements which exhibit a relatively low rationalization power of around 60%, this can mostly be explained by the simple dependence on the number of cationic ECN sites as presented in Section 4.2.1. The still relatively low rationalization power for S and Te might be explained by the formation of complex anions for which the concept of the coordination rule is less applicable. It might also just be an artifact of the small size of the respective subdatasets.

#### 4.2.3. Further possible influence factors

Apart from the aforementioned influences, more possible parameters exist for which their impact on the rationalization power of Beck’s extended CN rule is not yet fully understood. One of such possible factors is the symmetry of the coordination environments as already mentioned in George’s benchmark of Pauling’s rules (George *et al.*, 2020 ▸). It was shown that for oxides the fulfillment of Pauling’s electrostatic valence rule is much higher for very symmetric coordination environments. Since the electrostatic valence rule is closely related to the concept of the electrostatic imbalance *Y*, a similar behavior is expected for the extended CN rule. This was tested for ternary fluorides by assigning coordination polyhedra to all coordination environments using the software *Polynator* (Link & Niewa, 2023 ▸). The dataset was then limited to compounds in which at least one cation is coordinated by anions forming an undistorted platonic octahedron. This procedure yielded 81 structures of which all were rationalizable using the SNS. This exceptional agreement corresponds well with George’s findings (George *et al.*, 2020 ▸). However, the concept of the SNS is based on the minimization of the segregation index Σ and is therefore only indirectly linked to the electrostatic imbalance *Y* and thus Pauling’s electrostatic valence rule. That means that a minimal value for the segregation index does not always correspond to a minimal value for the electrostatic imbalance. Yet this is the case for the 81 structures containing platonic octahedra as all of those coordination matrices reach values of 0 for both Σ and *Y*. Segregation indices of 0 mean that all anions show the same coordination and the matrix therefore only contains one unique row. This is the case when the average CN *r* as well as all cationic contributions, *e.g.r*_A_ and *r*_B_, are integers. Since a cation in an octahedral coordination environment shows a CN of 6, all other CNs and the chemical index of the anions must be divisible by 3, 2 or 1 to yield integer results. Since the chemical index of the fluoride anions cannot assume a value of 1 for a ternary compound without mixed positions, this option can be excluded. A value of 2 is also unlikely, as it could only be achieved in compounds in which both cations show charges of +1. This means the other cations most likely show CNs of 3, 6, 9 or 12. Many of these structures are related to the perovskite type. For undistorted cuboctahedral coordination environments the same trend is observable, since all structures which contain cuboctahedra also contain octahedra. Only seven compounds contain undistorted, platonic tetrahedra, but all of these structures show deviations from Beck’s rule and therefore do not agree with the aforementioned behavior.

**Figure 13:**
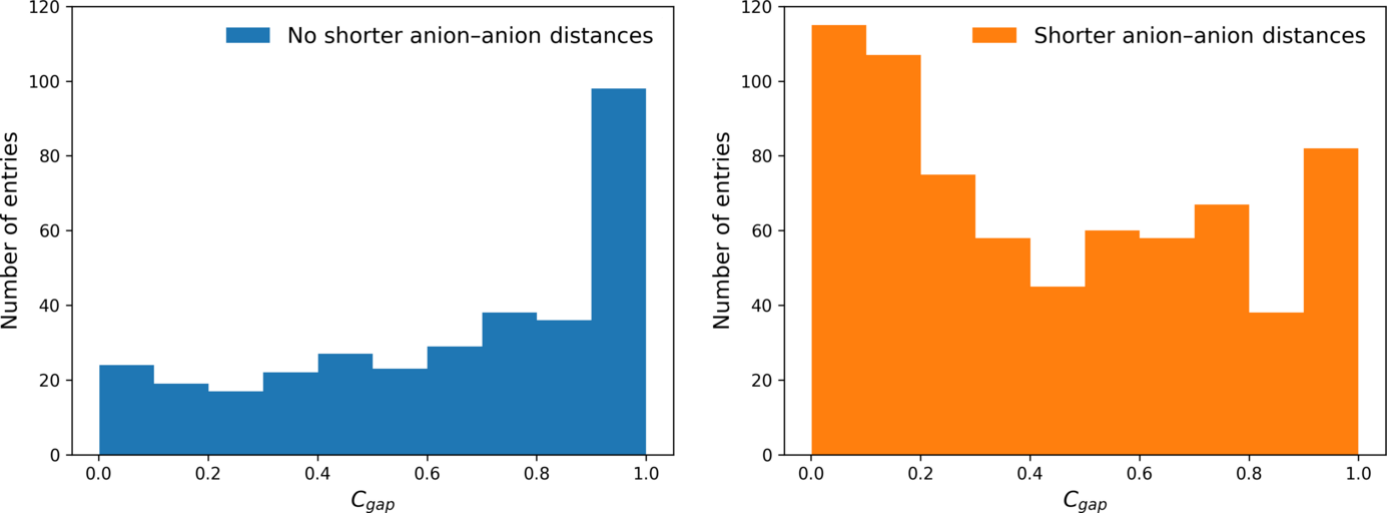
Histograms of the *C*_gap_ values for the subdatasets containing no anions closer to each other than to the central cation (left, 333 structures) and structures that exhibit this feature (right, 705 structures). The width of the bins was calculated using the Freedman–Diaconis rule for the subdataset without shorter anion–anion distances.

Another possible influence factor on the agreement is the ratio of the distances between cation and anion and two anions. Many structures exhibit coordination environments in which the distance from a coordinating anion to the central cation is larger than the distance to the next anion. For the 705 compounds which show this behavior the agreement between the coordination in the experimentally observed structure and the SNS drops to 50.6% whereas it rises to 79.6% for the 333 compounds not showing this feature. Interestingly, the 81 structures containing perfect octahedra 64 exhibit such a shorter anion–anion distance, which contradicts the previously discussed influence of the symmetry. This discrepancy can be traced back to the well definedness of the coordination sphere. In many structures it is not possible to assign the CNs unambiguously since there are no large gaps in the histogram of interatomic distances. This can be quantified by calculating the ratio of the of the second largest gap *d*_gap,2_ divided by the largest gap *d*_gap,1_ and subtracting it from 1, yielding the gap confidence *C*_gap_:
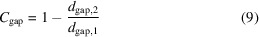


Calculating the *C*_gap_ values for the structures containing anions closer to each other than to the central cation as well as for the structures not showing this feature yields the histograms shown in Fig. 13 ▸. The histogram of the *C*_gap_ for the structures not featuring shorter anion–anion distances shows a tendency for high gap confidence values close to 1. This is a clear difference to the histogram of the gap confidence values for the structures exhibiting this feature, which tend to show lower *C*_gap_ values. All of the 81 octahedra containing structures show a gap confidence of 1, meaning that no other gap was found within the allowed CN range of 3 ≤ CN ≤ 12. This might explain the extraordinarily high agreement for these structures despite containing anions that are closer to each other than to the central cation.

## 5. Conclusion

In this work an assessment of the rationalization power of the extended CN rule was presented. This was achieved utilizing a full automatization of the application of Beck’s rule as shown in Fig. 6 ▸. For 59.9% of the 1038 compounds investigated there is a complete agreement between the coordination matrix of the experimentally determined coordination matrix and the SNS. For further 22.3% the deviation between the experimentally determined structure and the SNS could be explained using the electrostatic imbalance. This sums to a total rationalization power of 82.2%. With respect to the simplicity of Beck’s rule, this is a high value, which exceeds the rationalization power of each single Pauling rule as benchmarked by George *et al.* (2020 ▸). (For the third rule this claim is only true if compounds containing coordination polyhedra with shared edges qualify for disagreement.

This could make the extended CN rule a powerful tool to understand crystal chemistry and search for new functional materials. The latter would require Beck’s rule to not only hold descriptive but also predictive power. While the scope of this publication aims only to prove the descriptive capabilities of the rule, possible predictive powers will be subject to future investigation. Subsequent work also includes the mitigation of the still present limitations, mostly the exclusion of compounds containing unordered sites or multiple anions. The compounds that still disagree with the extended CN rule will be studied further, as they might show chemical peculiarities. Lastly the assessment of Beck’s rule will be extended to other (less ionic) compound classes (*e.g*. oxides, other halogenides).

## Figures and Tables

**Table 1:** Summary of the rationalization results obtained by automatic application of Beck’s extended CN rule to a dataset consisting of 1038 ternary fluorides

	Subgroup	No. of structures	Relative size (%)
Agreement of the element-specific coordination matrix	Correct	653	62.9
	Incorrect	385	37.1
Agreement of the ECN-specific coordination matrix	Correct	622	59.9
	Incorrect	416	40.1
Ratio of the electrostatic imbalances	*Y*_tot_ < *Y*_num_	229	22.1
	*Y*_tot_ * = Y*_num_	2	0.2
	*Y*_tot_ > *Y*_num_	185	17.8

## Data Availability

The code to reproduce the results can be accessed via https://github.com/Niklas-Langer/ExtendedCoordinationNumberRule-Publication.

## Footnotes

1 Some authors suggest CNs of 9 for both strontium ions (Von der Mühll, 1973 ▸). For this example, CNs of 9 and 10 are used. Both options yield the same results with respect to the crystal-chemical calculations presented in the following sections, since in both cases the simplest numerical solution does not agree with the experimentally determined crystal structure. However, they exhibit lower values for the electrostatic imbalance. This means they belong to the same group of entities as shown in Table 1 ▸.

2 This is due to the fact that the extended CN rule does not allow for fractional CNs (as *e.g.* proposed in the ECoN method; Hoppe, 1979 ▸). Since a homogenous distribution of ions also means that only a few different coordination environments exist for the anions, this concept is linked to Pauling’s fifth rule (rule of parsimony) (Pauling, 1929 ▸).

3 For some structures a coordination matrix different from the SNS can also achieve the same minimal segregation index as the SNS. This behavior is discussed in Section 4.1.

4 Despite its simplicity, the reciprocal largest gap algorithm still showed a decent performance in literature benchmarks, being able to identify the coordination environments of ternary compounds correctly in over 90% of the tested crystal structures (Pan *et al.*, 2021 ▸).

5 For the structures with the collection codes 5006 (Rb_3_CeF_7_), 15793 (Cs_3_CeF_7_) and 38300 (Cs_3_TbF_7_) the structures listed in the publications and in the ICSD do not account for the rotational disorder of the *M*F_7_^3−^ (*M* = Ce, Tb) groups present in the assigned (NH_4_)_4_ZrF_7_ type. The structures with collection codes 25428 (NaCrF_3_) and 47119 (Cs_6_Ni_5_F_16_) both miss one fluoride site. For the structure with the collection code 36078 (RbPaF_6_) the atomic coordinates of the protactinium ions are given as (1/4 1/4 0) instead of the correct (1/4 0 0).
